# Deletion of Mesenchymal Glucocorticoid Receptor Attenuates Embryonic Lung Development and Abdominal Wall Closure

**DOI:** 10.1371/journal.pone.0063578

**Published:** 2013-05-16

**Authors:** Aiqing Li, Rowan Hardy, Shihani Stoner, Jan Tuckermann, Markus Seibel, Hong Zhou

**Affiliations:** 1 Bone Research Program, ANZAC Research Institute, University of Sydney, Sydney, Australia; 2 Centre for Endocrinology, Diabetes and Metabolism, Institute of Biomedical Research, University of Birmingham, Birmingham, United Kingdom; 3 Institute of General Zoology and Endocrinology University of Ulm, Ulm, Germany; 4 Dept of Endocrinology & Metabolism, Concord Hospital, Sydney, Australia; Children’s Hospital Los Angeles, United States of America

## Abstract

As a member of the nuclear hormone receptor superfamily of ligand-activated transcription factors, the glucocorticoid receptor (GR) is essential for normal embryonic development. To date, the role of mesenchymal glucocorticoid signaling during development has not been fully elucidated. In the present study, we investigated the role of the GR during embryogenesis specifically in mesenchymal tissues. To this aim, we crossed GRflox mice with Dermo1-Cre mice to generate GR^Dermo1^ mice, where the GR gene was deleted within mesenchymal cells. Compared to their wild type littermates, GR^Dermo1^ mice displayed severe pulmonary atelectasis, defects in abdominal wall formation resulting in intestinal herniation, abnormal extracellular matrix synthesis in connective tissues and high postnatal lethality. Lungs of GR^Dermo1^ mice failed to progress from the canalicular to saccular stage, as evidenced by the presence of immature air sacs, thickened interstitial mesenchyme and an underdeveloped vascular network between E17.5 and E18.5. Furthermore, myofibroblasts and vascular smooth muscle cells, although present in normal numbers in GR^Dermo1^ animals, were characterized by significantly reduced elastin synthesis, whilst epithelial lining cells of the immature saccules were poorly differentiated. A marked reduction in normal elastin and collagen deposits were also observed in connective tissues adjacent to the umbilical hernia. This study demonstrates that eliminating the GR in cells of the mesenchymal lineage results in marked effects on interstitial fibroblast function, including a significant decrease in elastin synthesis. This results in lung atelectasis and postnatal lethality, as well as additional and hitherto unrecognized developmental defects in abdominal wall formation. In addition, altered glucocorticoid signaling in the mesenchyme attenuates normal lung epithelial differentiation.

## Introduction

As a member of the nuclear hormone receptor superfamily of ligand-activated transcription factors, the glucocorticoid receptor (GR) appears to play an essential role during development [1], [2], [3], [4]. During murine embryogenesis, the GR is constitutively expressed in a tissue and time-specific pattern in almost all tissues and organs that derive from the germ layers, including the endoderm, mesoderm and ectoderm [5], [6]. The lungs were shown to be one of the most important glucocorticoid target tissues during embryogenesis, with lung atelectasis resulting in post natal lethality in the global GR null mouse [4]. In these animals, a generalized increase in cellular proliferation was observed throughout the lung, whilst apoptosis remained normal [7]. Bird et al. hypothesized that this increase in cellular proliferation contributes to mesenchymal thickening, resulting in lung atelectasis.

Supporting these findings, the expression of the GR was shown to be markedly elevated during the canalicular and saccular stages of lung development in both human and animal models [4], [8], [9], [10], [11]. The importance of glucocorticoids in lung development and maturation is further demonstrated by their beneficial therapeutic actions in the treatment of respiratory distress syndrome in preterm infants. Whereas the role of the GR in lung development is undisputed, conflicting results arise from studies eliminating the GR conditionally in epithelial cells [12]. Manwani and colleagues describe an essential role of the GR in epithelial cells, whilst Habermehl and colleagues suggest a more critical role of the GR in mesenchymal cell types [10], [11]. In order to unequivocally eliminate the GR specifically in cells derived from mesoderm-derived tissue and to gain deeper insight into the consequences on lung development, we generated a mesenchymal GR conditional knockout mouse (GR^Dermo1^) by utilizing Dermo1-Cre transgenic mice. Dermo1 is a transcription factor belonging to the basic helix-loop-helix (bHLH) family and is highly expressed in mesoderm tissues during embryogenesis [13]. The Dermo1-Cre strain was created by a homologous knock-in of Cre into the Dermo1 gene locus, which allows for more precise expression of Cre in locations where Dermo1 is normally expressed under the endogenous promoter [13].

In the present study we found mice lacking GR in mesenchymal cells display neonatal lethality due to abnormal lung development. In addition, more than half of GR^Dermo1^ embryos presented with a defect in ventral abdominal wall formation. These results demonstrate that mesenchymal GR signaling plays a critical role in embryonic lung and the abdominal wall development.

## Materials and Methods

### Generation of Mice with Mesenchymal-specific Deletion of the GR Gene

Dermo1-Cre transgenic mice (C57BL/6 background) were generated as described previously [14], kindly provided by Dr. Orniz, (Washington University School of Medicine, St Louis, USA). GR floxed (GR^fl/fl^) mice were backcrossed to the C57BL/6 background for at least 5 generations as previously described [15]. For conditional inactivation of GR in mesenchymal cells, mice were generated with the genotype Dermo1^Cre+^;GR^fl/fl^ (referred to as GR^Dermo1^) by mating Dermo1^Cre+^;GR^fl/+^ mice with GR^fl/fl^ mice. The genotype Dermo1^Cre−^;GR^fl/fl^ and Dermo1^Cre−^;GR^fl/+^ littermates showed no phenotypic differences from wild-type mice, therefore they served as wild type controls (referred to as WT). To detect the Cre activity, Dermo1-Cre transgenic mice were also crossed with Rosa26R (R26R) reporter mice in which the LacZ gene expression can be activated by Cre-mediated recombination [14]. Mice were maintained at the animal facilities of the ANZAC Research Institute, Sydney, Australia, in accordance with institutional animal welfare guidelines and an approved protocol.

### Primary Fibroblast Culture

New born pups were decapitated and eviscerated and the skin dissected from the carcass. After washing with PBS to remove any blood, the carcass and skin were minced with a scalpel blade. Dissociated tissue mixture and skin were incubated in culture media containing 0.25% trypsin, 0.1% collagenase type 4 (Worthington Biochemical Corp, Freehold, NJ, USA) and 400ug/ml deoxyribonuclease I (Worthington Biochemical Corp, Freehold, NJ, USA) and shaken vigorously for 30 min at 37°C. Digested tissues were pressed through a 70 µm nylon cell strainer and centrifuged at 1200 rpm for 3 min. The tissue pellet was resuspended in high glucose DMEM containing GlutaMAX (Life Technologies, NY, USA) and supplemented with 10% heat-inactivated FBS, 100 U/ml penicillin and 100 mg/ml streptomycin and cultured at 37°C, 5% C02. Culture media was changed every three days and cells subcultured at 80 to 90% confluence prior to characterization at passage two.

### X-gal Staining

X-gal staining of Dermo1^Cre+/−^;R26R^+/−^ mice was performed in embryos. Briefly, tissue were collected and fixed with 0.2% glutaraldehyde and 2% paraformaldehyde in PBS, pH 7.4 and processed for frozen sections. Sections were rinsed with PBS containing 2 mM MgCl2 and 0.01% NP40 and stained with X-Gal staining solution (2 mM MgCl2, 0.01% NP40, 5 mM potassium ferricyanide, 5 mM potassium ferrocyanide and 1 mg/ml X-Gal in PBS) for 16 hr at 37°C in the dark. All sections were counterstained with Nuclear Fast Red.

### Histological Staining and Immunohistochemistry

Embryos were isolated at E14.5, E16.5, E17.5 and E18.5 from a minimum of three different litters. Lung tissues were dissected and fixed in 4% paraformaldehyde, paraffin embedded, and sectioned at 5 µm. Sections were stained with hematoxylin and eosin (H&E) for histological analysis. Quantification of fibronectin positive cells in a 50 ^µm2^ area was determined by immunohistochemistry in the distal lung of 5 WT and 5 GR^dermo1^ mice. Positively stained cells were analyzed using Image J software. Masson and PAS staining (Sigma-Aldrich) were performed according to kit instructions. For elastin staining, sections were stained with Resorcin-fuscin solution (Electron Microscopy Sciences, Hatfield, PA) and were counterstained with Tartrazine (Sigma-Aldrich). For immunohistochemistry, antigen retrieval was achieved by immersing sections in 10 mM citric acid at 95°C for 45 minutes. Vessel diameter and number were calculated following α-smooth muscle actin staining, using Image J in matched sections of whole lung. The following rabbit polyclonal antibodies and dilutions were used: pro-SPC (Chemicon, AB3786, 1∶2000), AQP5 (Abcam, ab78486 1:500), CD31 (Abcam, ab28364, 1∶50), fibronectin (Sigma, F3648, 1∶400), α-smooth muscle actin (Abcam, ab5694, 1∶200), GR (Santa Cruz Biotechnology, sc-1004, 1∶200), GILZ (Santa Cruz Biotechnology, sc-33780, 1∶200). The signals were detected using biotinylated goat anti-rabbit secondary antibodies (Vectastain ABC-Peroxidase Kits, Vector Laboratories, Burlingame, CA, USA) and DAB (Vector Laboratories). Sections were counterstained with Harris Hematoxylin.

### MRI

Embryos were fixed in 4% PFA for 7 days. PFA was changed every 48 hr. Embryos were then embedded in 1% agarose (Invitrogen) containing 2 mM Magnevist (Bayer). MRI was performed as described by Schneider et al., [16].

### Lung Histomorphometric Analysis

Proportions of lung tissues/airspaces were assessed in H&E stained sections (x400 magnification) of E16.5 and E18.5 embryos. Measurements were performed using NIH Image J software.

### Quantitative RT-PCR

Total RNA from lung was isolated by Trizol reagent (Invitrogen) and further purified by RNeasy mini kit (Qiagen) according to the manufacturer’s instructions. First strand cDNA was synthesised from 1 µg of total RNA SuperScript^TM^III Reverse Transcriptase (Invitrogen) following oligo (dT) priming. Real Time RT-PCR was carried out using iQ™ SYBR Green Supermix (Bio-Rad) and amplifications were performed on a iCycler iQ5 Real-Time PCR Detection System (Bio-Rad). 18S was used for cDNA normalization. Primer sequences used are summarized in Table 1.

**Table 1:** Primer pairs used for real-time RT-PCR.

Gene	Fwd	Rvs
**18S**	**CATGATTAAGAGGGACGGC**	**TTCAGCTTTGCAACCATACTC**
**Elastin**	GCTACTGCTTGGTGGAGAATG	CCCTTGGAGATGGAGACTGT
**T1a**	ATGAATCTACTGGCAAGGCAC	CCATCTTTCTTATCTGTTGTCTGC
**SPA**	TTCAAGGCAAACACGGTG	CCTCAGTGATGTAAAGTGGACG
**SPB**	AGTGGCTACTGCTGCTTCCTA	ATCTTCCTTGGTCATCTTTGTGA
**AQP5**	CAGACCTCAGAGATTGTGAAGG	CAGAAATAAATAAGATGGCACTCG
**11b-HSD1**	GGAGCCGCACTTATCTGAA	GACCTGGCAGTCAATACCA
**Collagen T1**	CCAGTGGCGGTTATGACTT	GCTGCGGATGTTCTCAATC

### Statistical Analysis

Results are presented as mean±SEM. Differences between groups was analyzed by unpaired students T test. Differences were considered statistically significant when p<0.05.

## Results

### High Neonatal Lethality in Postnatal GR^Dermo1^ Mice

**Figure 1:**
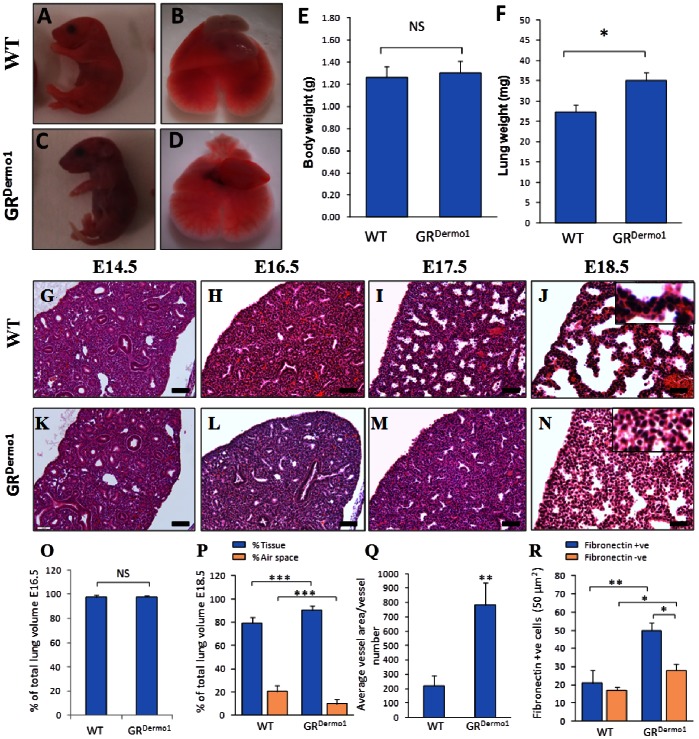
Postnatal lethality and impaired lung sacculation in GR^Dermo1^ mice. Gross morphology of pups (A,B) and whole lung specimens (C,D) of WT and GR^Dermo1^ mice. Average total body weight (E) and total lung weights (F) in WT and GR^Dermo1^ mice. H&E staining of distal pulmonary sections of WT and GR^Dermo1^ lung at E14.5 (G,K), E16.5 (H,L), E17.5 (I,M) and E18.5 (J,N). Inserts in J and N show increased magnification of the mesenchyme lining the saccules. Average total lung volume of WT and GR^Dermo1^ mice measured at E16.5 (O) and E18.5 (P). Average vessel diameter (Q) in WT and GR^Dermo1^ pulmonary tissues. Fibronectin positive/negative cells (R) in WT and GR^Dermo1^ lung in a 50 µm^2^ area. Data shown are either representative of, or the combined means of 9 WT and 9 GR^Dermo1^ mice. Magnification 20X, Scale bar 50 µm. *p<0.05, **p<0.005, ***p<0.0005.

Of a total of 223 GR^Dermo1^ mice, only 2.7% survived beyond weaning. As GR^Dermo1^ embryos (E14–E18) had an expected Mendelian segregation, targeted mesenchymal GR deletion appears to result in postnatal lethality. Indeed, newborn GR^Dermo1^ mice presented with cyanotic skin (Fig. 1A–B) and died shortly after birth. Compared to their age-matched WT littermates, lungs from GR^Dermo1^ mice were pale with an underdeveloped vascular network (Fig. 1C–D). Average lung weights of GR^Dermo1^ pups were significantly heavier than those of WT littermates (35.1±6.0 mg, n = 7 versus 27.4±5.7 mg, n = 9, p = 0.02), whilst no significant difference was observed in total body weight (Fig. 1E–F, Fig. S1A). In addition to the pulmonary phenotype, 37.5% of GR^Dermo1^ newborn pups presented with a peri-umbilical anterior abdominal wall defect and intestinal herniation, which was, however, unassociated with postnatal lethality. These findings suggested that deletion of the mesenchymal GR leads to neonatal lethality due to abnormal lung development.

### Defective Alveolar Sacculation and Intestinal Herniation in Prenatal GR^Dermo1^ Mice

To further characterize the presumed defect in pulmonary development, the lungs of embryos at E14.5, E16.5, E17.5 and E18.5 were examined in detail. GR^Dermo1^ embryos at both E14.5 and E16.5 exhibited normal pulmonary structures, similar to that of their age-matched littermate controls (Fig. 1G, H vs. K, L). From E17.5 to E18.5, lungs from WT animals progressed to the saccular stage, as characterized by well-formed saccules, larger air spaces and thinning of the interstitial mesenchymal tissue (Fig. 1I–J). In contrast, lungs from GR^Dermo1^ embryos remained in the canalicular phase, displaying small immature air sacs and a thickened mesenchyme (Fig. 1M–N). At E16.5, the ratio of lung tissue to alveolar space was similar in GR^Dermo1^ and WT animals (97.7% vs 98.0%) (Fig. 1O), whilst at E18.5, this ratio had changed to be significantly greater in GR^Dermo1^ than in WT mice (90.3% vs 79.6% p<0.001) (Fig. 1P).

**Figure 2:**
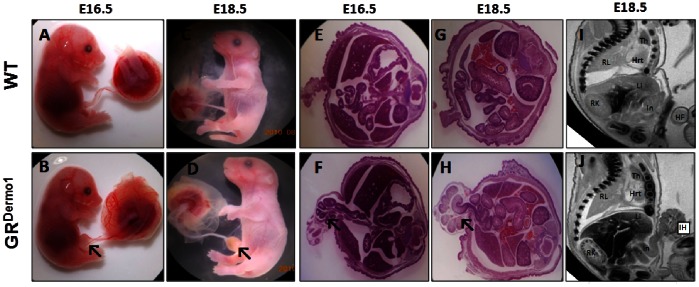
Intestinal herniation in GR^Dermo1^ mice. Gross morphology of WT and GR^Dermo1^ fetuses at E16.5 (A,B) and E18.5 (C,D). H&E staining of WT and GR^Dermo1^ ventral wall defects at E16.5 (E,F) and E18.5 (G,H). Magnetic resonance imaging of the abdominal region of WT and GR^Dermo1^ mice, showing the ventral wall defect at E18.5 in GR^Dermo1^ animals (I,J). Results shown are representative of 9 WT and 9 GR^Dermo1^ mice. Arrows indicate intestinal herniation. RL = Right lung, RK = Right Kidney, IH = Intestinal Herniation, Hrt = Heart, L = Liver, I = Intestines, Th = Thyroid.

**Figure 4:**
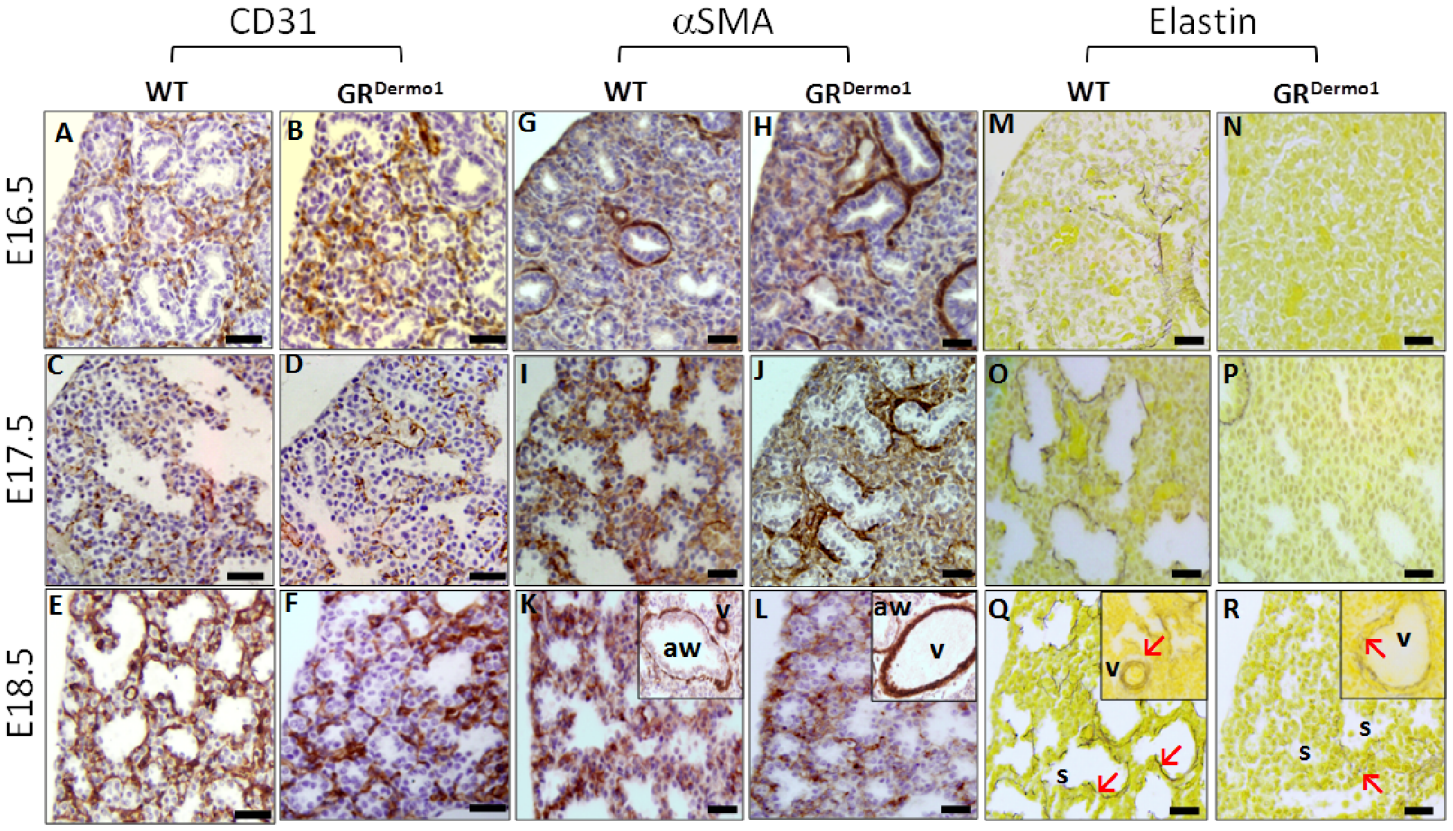
Expression of mesenchymal markers and elastin at E16.5, E17.5 and E18.5. IHC staining for CD31 (A–F) and αSMA (G–L) in pulmonary sections of WT and GR^Dermo1^ mice at E16.5, E17.5 and E18.5. Hart stain for elastin deposition (M–R) in pulmonary tissues of WT and GR^Dermo1^ animals at E16.5, 17.5 and 18.5. Insert in K and L show increased magnification of conducting airways and vessels. Insert in Q and R show increased magnification of vessels. All results are representative of 5 WT and 5 GR^Dermo1^ mice. Aw = conducting airway; v = vessel; s = saccule; **↖ = **positive staining. Scale bar 20 µM.

At E18.5 clear differences in pulmonary artery structure were visible by histology. Pulmonary arteries were identified by the presence of thickened αSMA positive smooth muscle walls lining vessels (Fig. 4K–L inserts). Pulmonary blood vessels in GR^Dermo1^ embryos at E18.5 were visibly less well developed, being significantly larger in diameter than in WT mice (218.1±71.1 vs 784.4±151.4, average vessel area/vessel number; p<0.05) (Fig. 1Q). At the same time, interstitial (inter-alveolar) mesenchymal thickness was greater in GR^Dermo1^ embryos than in WT littermates. This was confirmed by fibronectin staining, which demonstrated significantly greater numbers of interstitial fibroblasts in GR^Dermo1^ mice at E18.5 compared to the lungs of WT embryos (50.1±4.2 vs 21.1±7 cells/50 µm^2^; P<0.005; Fig. 1R). These data indicate that mesenchymal deletion of the GR results in an arrest of pulmonary development at the canalicular stage. In addition to the pulmonary phenotype, more than half of GR^Dermo1^ embryos (9/17 at E16.5; 14/27 at E18.5) presented with a defect in ventral abdominal wall formation, which resulted in significant intestinal herniation (Fig. 2A–D). Externalization of the intestines was clearly visible by both H&E staining and magnetic resonance imaging (MRI) at E16.5 and E18.5 (Fig. 2E–H). To determine if any additional organs were affected, GR^Dermo1^ mice were studied by conventional histology and MRI. Examination of multiple organs, including the, brain, heart, kidney, liver, thymus and placenta demonstrated no further abnormalities (Fig. 2I–J). The abnormalities in ventral body wall formation closely correlated with an increasing severity of the lung phenotype in GR^Dermo1^ mice (Fig. S1A). Consequently, GR^Dermo1^ mice presenting with both the pulmonary phenotype and intestinal herniation were used for further characterization.

### Mesenchymal GR Expression is Up-regulated During E17.5 in WT but not GR^Dermo1^ Mice

**Figure 3:**
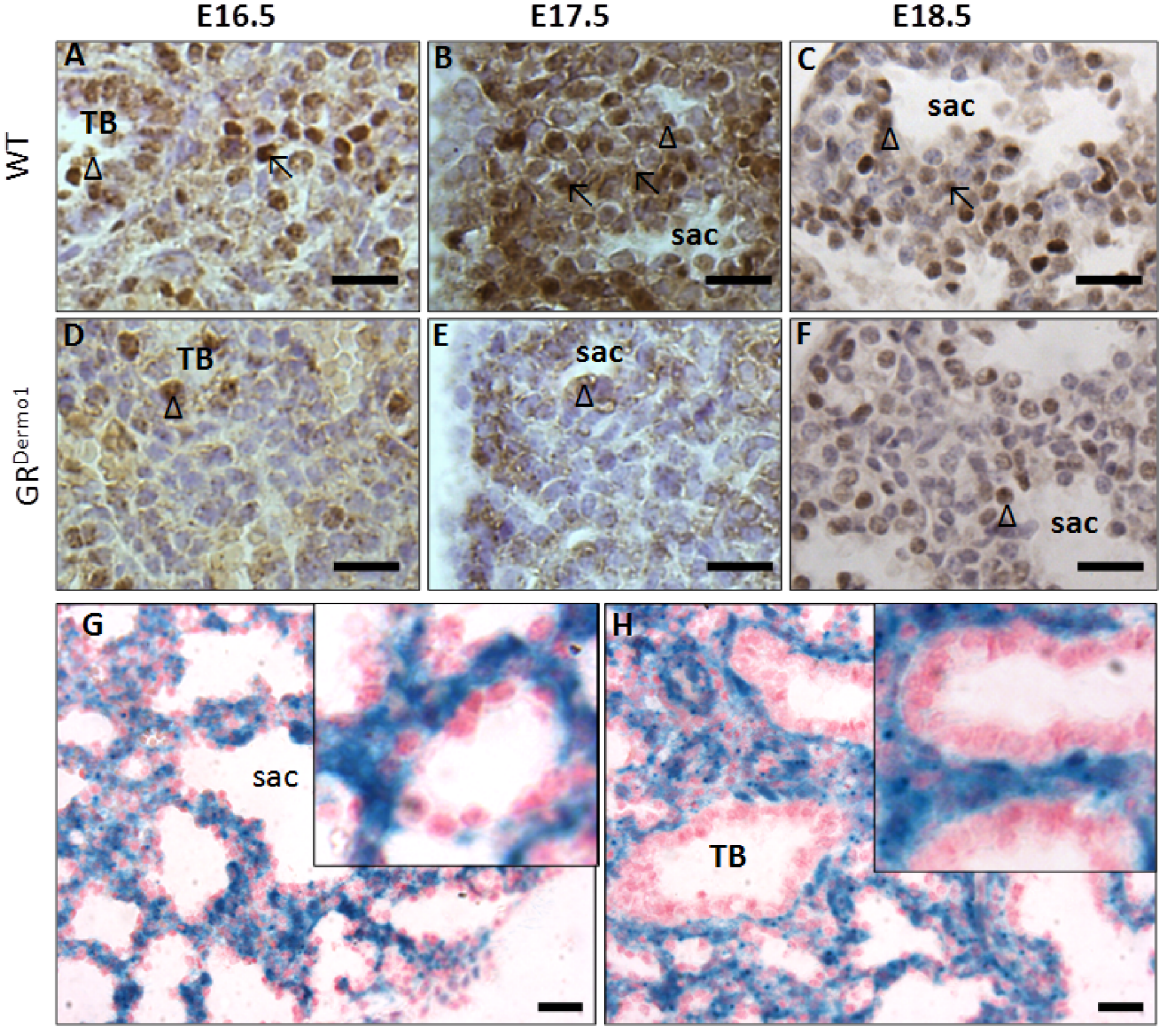
Confirmation of conditional GR KO. IHC staining for GRα in pulmonary sections of WT and GR^Dermo1^ mice at E16.5 (A,D), E17.5 (B,E) and E18.5 (C,F). X-gal stain was performed for Cre activity in the lung from E18.5 Dermo1^Cre+/−^;R26R^+/−^ mice in distal (G) and proximal (H) airways. Inserts in G and H show increased magnification of the mesenchyme lining saccules and terminal bronchioles respectively. Results shown are representative of 5 WT and 5 GR^Dermo1^ mice. ↖ = GR positive staining in mesenchyme; Δ = GR positive staining in epithelium; Sac = alveolar saccule; TB = terminal bronchiole. Scale bar, 10 µm.

In WT mice, GR expression was located throughout the pulmonary mesenchyme and epithelia between E16.5–18.5, and was greatly up-regulated at E17.5 (Fig. 3A–F). GR expression was entirely absent in the pulmonary mesenchyme of GR^Dermo1^ mice between E16.5 and E18.5. These data were supported by X-gal staining of Dermo1-Cre;Rosa26R mice, which strongly suggested that Dermo1-Cre recombination takes place in the mesenchymal compartment, while the GR remained intact in epithelial cells (Fig. 3G–H). This difference in pulmonary GR expression between WT and GR^Dermo1^ mice was most marked at E17.5, i.e. whilst GR expression was at its greatest within the mesenchymal compartment (Fig. 3B, E). Mesenchymal GR expression is significantly increased at E17.5 may suggest a key role for glucocorticoid signaling in lung development progressing from canalicular stage to secular stage.

### Pulmonary Interstitial Fibroblast Function is Defective in GR^Dermo1^ Mice

Mesenchymal-derived cells within lung tissue include interstitial fibroblasts, myofibroblasts, smooth muscle and vascular endothelial cells. To define which mesenchymal cell subpopulations contribute to the distal lung phenotype in GR^Dermo1^ animals, we performed selective stains for interstitial fibroblasts (fibronectin), myofibroblasts, smooth muscle cells (α-SMA), and vascular endothelial cells (CD31). Quantification of IHC staining for the stromal marker fibronectin showed significantly greater numbers of pulmonary interstitial fibroblasts in GR^Dermo1^ compared to WT mice (50.1±6.9 vs 21.2±4.2 cells/50 µm^2^; P<0.005) (Fig. 1R; sup Fig. 1b). These data indicate that the increased cell populations observed in the lungs of GR^Dermo1^ animals are of mesenchymal origin. This prompted us to examine markers of specific mesenchymal subpopulations. CD31 staining for endothelial cells in WT lungs between E16.5 and E18.5 was concentrated around the lining of immature and well formed saccules, supporting the role of the endothelium in gas exchange in these structures (Fig. 4A,C,E). Other than the positive endothelial CD31 staining observed in immature vessels adjacent to the saccules, CD31 staining was absent in the mesenchyme. Similar CD31 staining was seen in lungs of E16.5–E18.5 GR^Dermo1^ mice, although as these tissues resembled lung from the canalicular phase, this was mostly observed lining immature alveolar saccules (Fig. 4B,D,F). Closer examination of αSMA, to identify myofibroblasts and smooth muscle cells, demonstrated comparable staining throughout the mesenchyme, bronchioles and vessels in lung tissues from both GR^Dermo1^ and WT mice, which increased significantly from E17.5 onwards (Fig. 4G–L). Despite our reported differences in vessel size, closer examination of the staining patterns in smooth muscle of vessels and bronchioles were identical between GR^Dermo1^ and WT mice (Fig. 4K–L). In the absence of a significant variation in numbers, or patterns of mature mesenchymal cell staining, we went on to examine the functional maturity of interstitial fibroblasts and myofibroblasts by examining their elastin production. Normally, these cells generate abundant amounts of elastin, which is required for normal saccular progression and maturation [17]. At E16.5, lungs from both GR^Dermo1^ and WT mice displayed minimal elastin staining, with sparse deposits surrounding the bronchioles (Fig. 4M–N). In lungs taken from WT animals at E17.5 and E18.5, intense staining for elastin deposits was observed throughout the mesenchymal tissues. In particular, strong elastin staining was observed lining mature alveolar saccules, whilst a double layer of staining was seen around well formed small vessels (Fig. 4O,Q). By comparison, elastin deposits were either significantly reduced or absent in lungs taken from GR^Dermo1^ mice within the mesenchymal tissue and saccules, whilst staining in the abnormal enlarged vessels was discontinuous, lacking the double layer (Fig. 4P,R). Within whole tissue homogenates of lung, elastin mRNA expression was shown to be significantly reduced in GR^Dermo1^ mice relative to their wild type counterparts (19.2 fold; p<0.05) (Fig. S1C). These data suggest an important role for glucocorticoid signaling in the regulation of elastin synthesis by interstitial fibroblasts, myofibroblasts and smooth muscle cells. Defective elastin synthesis or attenuated functional maturity of myofibroblasts may contribute to the delayed saccular progression and vessel formation observed in the developing lungs of GR^Dermo1^ mice.

### Defective AECI and AECII Differentiation in E18.5 GR^Dermo1^ Mice

**Figure 5:**
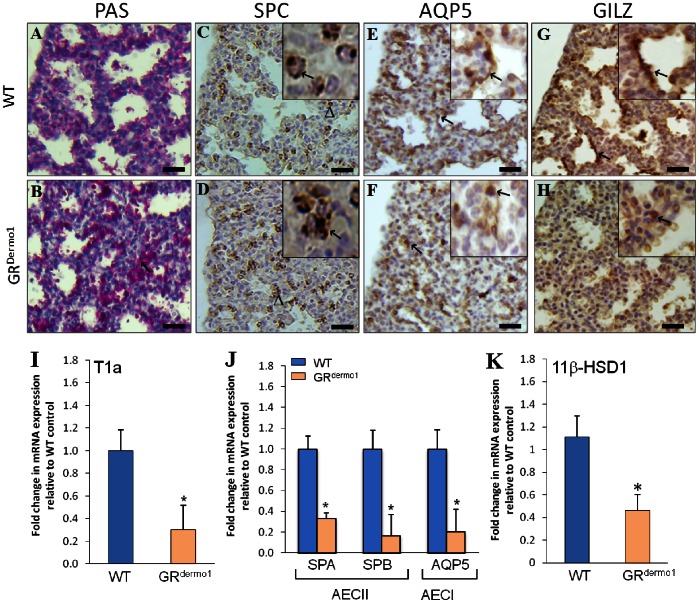
Defective AECI and AECII differentiation in GR^Dermo1^ mice. PAS stain for undifferentiated alveolar epithelial cells (A,B) in lung tissues of WT and GR^Dermo1^ at E18.5. IHC staining for SPC (C,D), AQP5 (E,F) and GILZ (G,H) in pulmonary sections taken from WT and GR^Dermo1^ animals at E18.5. Inserts in C and D show increased magnification of intracellular SPC staining within epithelial cells. Inserts in E and F show increased magnification of AQP5 staining in epithelial cells lining saccules. mRNA expression of T1a (I), SPA, SPB, AQP5 (J) and 11β-HSD1 (K) in GR^Dermo1^ lung homogenates relative to WT control. All results are either representative or the means of 5 WT and 5 GR^Dermo1^ mice. ↖ = SPC and AQP5 positive stain. *p<0.05. Scale bar 20 µM.

During embryogenesis, the murine pulmonary airways are lined with structurally and functionally different epithelial cell types. Thus, at the saccular stage, alveolar saccules consist of flat alveolar epithelial type I cells (AECI), cuboidal alveolar epithelial type II cells (AECII) and undifferentiated alveolar epithelial cells that are rich in cytoplasmic glycogen [18]. PAS staining at E18.5 confirmed the presence of significantly greater numbers of immature glycogen positive epithelial cells lining immature saccules in the GR^Dermo1^ lung relative to WT (Fig. 5A–B). Messenger RNA expression of the embryonic epithelial marker Podoplanin (T1a) and the specific markers for AECII (surfactant protein A (SPA) and B (SPB)) and AECI (Aquaporin 5 (AQP5) and Podoplanin (T1a)) were measured in whole lung homogenates to indicate numbers of their respective populations. Expression of the marker T1a (that stains all epithelial cells in embryonic tissues [19]) was significantly reduced in GR^Dermo1^ lung (3.3 fold; p<0.05) (Fig. 5I). Expression of both SPA and SPB, were significantly reduced in GR^Dermo1^ lungs relative to WT controls (3.0 and 5.9 fold; p<0.05) (Fig. 5J). Similar patterns were observed for AQP5, which was also significantly reduced in GR^Dermo1^ lung (4.7 fold; p<0.05) (Fig. 5J). These data were confirmed by IHC staining for SPC (for AECII) and AQP5 (AECI). Positive staining of SPC in mature, active AECII was concentrated within the granules of lamilar bodies in the cytoplasm of WT lungs at E18.5 (Fig. 5C). In contrast, SPC staining was consistently more diffuse within the cytoplasm of GR^Dermo1^ epithelial cells, indicating an abnormal morphology (Fig. 5D, insert). Staining for AQP5 was observed in mature flattened AECI, lining the saccules of lungs from WT mice as a thin continuous brown line around the surface of the saccules at E18.5 (Fig. 5E). By comparison, AQP5 staining was more frequently localized within cuboidal cells of lung tissue obtained from GR^Dermo1^ mice at E18.5, indicating these cells are immature (Fig. 5F). These results suggest that deletion of the mesenchymal-specific GR results in impaired AECI and AECII differentiation.

### Glucocorticoid Signaling is Attenuated in GR^Dermo1^ Lung Epithelia

As glucocorticoids have also been shown to play a key role in the differentiation of epithelia in fetal lung, we examined downstream glucocorticoid-signaling in pulmonary tissue of GR^Dermo1^ mice by measuring the expression of the glucocorticoid-induced leucine zipper (GILZ) [10], [11]. GILZ has been shown to be constitutively expressed within epithelial lining cells within the lung [20]. At E18.5 in WT lungs, GILZ staining was observed strongly in the cells lining the developing saccules (Fig. 5G). This pattern of staining closely co-localized with the AEC1 marker AQP5, indicating that glucocorticoid signaling is elevated within these epithelial cells. In contrast, despite normal expression of the GR within the epithelial compartment, GILZ expression was significantly attenuated within the epithelia of GR^Dermo1^ mice (Fig. 5H). In the presence of reduced glucocorticoid signaling in the epithelial compartment, we examined a candidate gene for local glucocorticoid metabolism in whole lung homogenates. mRNA expression of the glucocorticoid activating enzyme 11β-hydroxysteroid dehydrogenase (11β-HSD1) was significantly reduced in lung homogenates of GR^Dermo1^ mice compared to WT (2.5 fold; P<0.05) (Fig. 5K). These data suggest that local glucocorticoid activation may be attenuated within the lungs of GR^Dermo1^ mice, which could contribute to the reduced glucocorticoid signaling within the epithelial cells of these animals.

### Abdominal Wall Defects in E18.5 GR^Dermo1^ Mice

**Figure 6:**
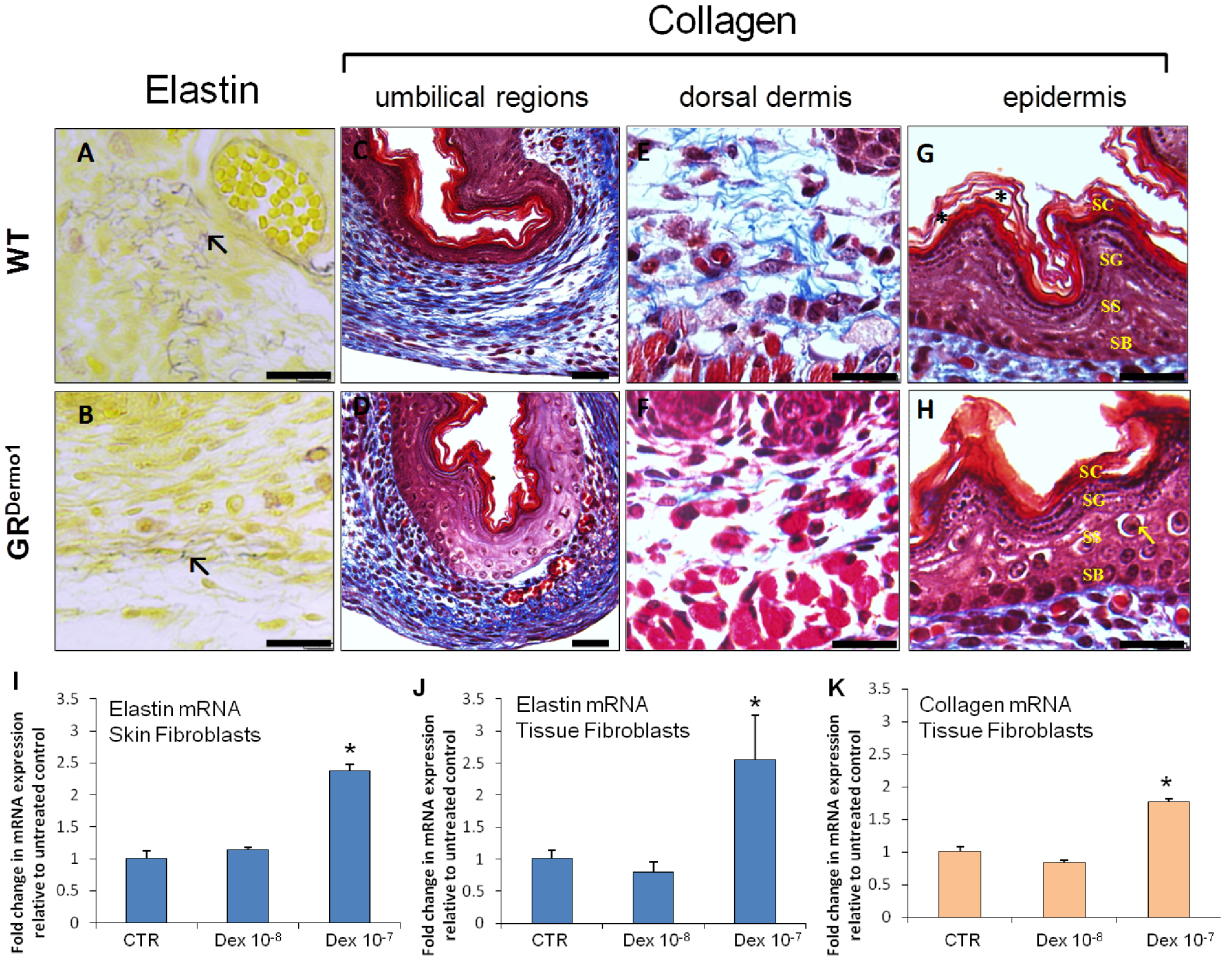
Defective elastin and collagen synthesis in connective tissue surrounding the intestinal herniation in GR^Dermo1^ animals. Hart stain for elastin deposition (A,B) in the unbilical region of WT and GR^Dermo1^ mice at 18.5. Masson stain for collagen fibers (blue) in the unbilical region (C,D), dorsal dermis (E,F) and epidermis (G,H) in WT and GR^Dermo1^ mice. Fold change in mRNA expression of elastin (I–J) and collagen (K) in fibroblasts isolated from skin and whole tissue homogenates, following treatment with dexamethasone (10 and 100 nmol/l) relative to untreated controls in wild type mice. All results are either representative or the means of 5 WT and 5 GR^Dermo1^ mice. Sc = stratum corneum; Sg = stratum granulosum; Ss = stratum spinosum; Sb = stratum basale; ↖ = elastin. *p<0.05. Scale bar: 20 µM.

Having identified a lack of elastin synthesis as being an important component of pulmonary maldevelopment in GR^Dermo1^ mice, we examined whether the same defect may play a parallel role in the pathogenesis of the ventral wall defect observed in many of these mice. In addition to elastin we also examined the expression of type I collagen, as both of these extracellular matrix components play a key role in maintaining the structural integrity around the umbilical region. Once again, elastin staining was reduced in GR^Dermo1^ mice, with elastin deposits being less frequent or entirely absent in periumbilical interstitial tissues (Fig. 6A–B). Masson stain for collagen identified that the periumbilical collagen matrix was disorganized in GR^Dermo1^ mice, lacking the organized longitudinal fiber bundles seen in WT mice (Fig. 6C–D). Within the dorsal dermis, staining for collagen fibers was significantly reduced in GR^Dermo1^ mice (Fig. 6E–F). To examine the role of glucocorticoid signaling in mesenchymal elastin and collagen synthesis more closely, we looked at the effects of glucocorticoids in primary fibroblast cultures isolated from newborn murine skin and whole tissue homogenates (Fig. 6I–K). Messenger RNA expression of elastin was shown to be positively regulated by the glucocorticoid dexamethasone at 100 nm in both skin fibroblasts and whole tissue homogenates (2.3 and 2.5 fold for skin and tissue respectively; p<0.05) (Fig. 6I–J). We observed positive regulation of type 1 collagen mRNA by glucocorticoids (1.8 fold; P<0.05) (Fig. 6K). As described in pulmonary tissues above, it appeared that epithelial cell differentiation was also disrupted within the epithelium. Examination of the stratum spinosum and stratum basale of the epidermis confirmed increased numbers of enlarged immature cells (Fig. 6G–H). These findings demonstrate that glucocorticoids regulate extracellular matrix synthesis in mesenchymal cells in a similar manner to that observed in the lung for elastin.

## Discussion

The present work demonstrates that the inactivation of the mesenchymal GR in mice results in postnatal lethality due to a failure of the embryonic lung to progress from the canalicular to the saccular stage of development. Furthermore, we show that the disruption of mesenchymal GR signaling results in hitherto unreported developmental abnormalities in abdominal wall formation due to defective extracellular matrix synthesis in connective tissues.

Whilst the lungs of WT animals progress through to the saccular phase between E17.5 and E18.5, lungs from GR^Dermo1^ mice remained in the canalicular phase, characterized by immature, poorly developed alveolar saccules and an undeveloped vasculature. We have confirmed that this failure in embryonic lung progression coincides with defective myofibroblast function, with abrogated glucocorticoid-regulated elastin synthesis. Elastin has been shown to play an important role in normal postnatal alveolarization and may contribute to the lung atelectasis observed in the GR^Dermo1^ mouse. Alternatively, defective elastin synthesis may be indicative of functionally immature myofibroblasts, and as such, representative of a loss in other extracellular matrix components required for embryonic alveolarization.

Finally, we have identified that the loss of glucocorticoid signaling in mesenchymal cells translates directly into cell-non-autonomous effects on alveolar epithelial cells, restricting their differentiation and resulting in an abnormal cellular morphology. This clearly demonstrates that mesenchymal GR expression is essential in the normal formation of alveolar saccules and vessels and that its absence is a major factor in the lethal phenotype of GR^Dermo1^ mice. Consequently, this study supports the importance of elevated pulmonary GR expression reported during the canalicular phase of lung development [5], [6].

### Defective Elastin Synthesis in Interstitial Fibroblast Populations

Having confirmed an important role for the mesenchymal GR in lung development, we further demonstrate that the precursor cell pool of interstitial fibroblasts was largely expanded in the lungs of GR^Dermo1^ mice at E18.5. Among the mesenchymal-derived cell populations, endothelial cells, smooth muscle cells and myofibroblasts all exhibited a normal morphology and similar patterns of staining in the lungs from GR^Dermo1^ mice compared to wild type animals. The only differences observed, occurred as a result of the immature canalicular morphology of the lung in the GR^Dermo1^ mice rather than a difference in cell numbers. These findings suggested that the phenotypes observed in the lungs of the GR^Dermo1^ mouse were not occurring as a result of the absence of these cell types.

Myofibroblasts are key producers of extracellular matrix components required for alveolarization. Elastin synthesis by interstitial fibroblasts and myofibroblasts in the lung is significantly up-regulated prior to the saccular stage of development, and has been shown to be critical for normal postnatal alveolarization and survival in mice [17], [21], [22]. In the GR^Dermo1^ mouse we identified a marked decrease in elastin synthesis throughout the lungs. However, myofibroblast numbers and their pattern of expression appeared normal in these animals, suggesting that these cells are not functionally active.

Analysis of elastin mRNA expression in interstitial fibroblasts from skin and whole tissue homogenates revealed it was positively regulated by the glucocorticoid dexamethasone. This may indicate that the positive transcriptional regulation of elastin by glucocorticoids is a generic effect of mesenchymal cells isolated from our model and suggests an important role for glucocorticoid signaling in normal ECM production by lung fibroblasts. This was further supported by observations of positive collagen mRNA regulation by glucocorticoids in our fibroblast cultures (Fig. 6K). To be truly robust these findings would need to be confirmed in mesenchymal cell populations isolated from embryonic lung between day E16.5 and 18.5. Unfortunately, to examine glucocorticoid ECM regulation in all the mesenchymal subsets of the lung went beyond the scope of this study. Despite this, whilst excepting these limiting factors, our experiments still provide valuable insight into elastin regulation by mesenchymal stromal cells.

This finding is further supported by previous studies confirming that elastin is a glucocorticoid target gene. Its promoter contains three GR response elements, all of which are positively regulated by glucocorticoids in interstitial fibroblasts of the neonatal lung [23], [24], [25]. Indeed, the physiological and therapeutic actions of glucocorticoids on pulmonary tissues are in part mediated via increased elastin synthesis by interstitial fibroblast populations [8], [17], [25], [26]. Despite this, the detrimental effects on alveolarization in the elastin null mouse do not become apparent until postnatal day 4 [22]. This would suggest that deficient elastin expression in the GR^Dermo1^ mouse is not the primary cause of defective embryonic sacculation in these animals. Instead, abrogated elastin synthesis may contribute to delayed saccular progression in the GR^Dermo1^ mouse or it may be indicative of a more general loss in myofibroblast functionality in the lung. Consequently, other key stromal extracellular matrix components such as collagen, fibulin-5 or fibrillin-1 may be disrupted in myofibroblasts, resulting in the observed defects in saccular progression and normal lung alveolarization.

This mechanism may also explain the underdeveloped vascular network in GR^Dermo1^ mice. Elastin plays a critical role in maintaining the structural characteristics of normal blood vessels [27]. Disrupted expression of elastin and other extracellular matrix components in the lungs of GR^Dermo1^ mice may contribute to the abnormal vascular phenotype observed in these animals. Unfortunately, these finding are complicated by the inability to dissect out local lung mesenchymal effects on pulmonary atelectasis from distant non-lung mesenchymal signaling that might influence lung development. Our data certainly support an important role for lung myofibroblasts, however, without specific GR KO targeting of mesenchymal cells of the lung, their relative contribution to the observed phenotype cannot be confirmed.

One question remaining is why the targeted deletion of elastin within mesenchymal cell subsets, which we have shown affect extracellular matrix synthesis, does not affect more organs. One explanation might be that any extracellular matrix defects are not yet fully apparent at this early stage of development, whilst the defects in the lung are most apparent due to their immediate role in post natal survival.

These findings have a number of parallels with previous work by Habermehl et al. where the role of mesenchymal GR signaling was examined using the GR^Col1−Cre^ [10]. This also resulted in postnatal lethality as a result of arrested lung development and atelectasis. This was correlated with enhanced epithelial and mesenchymal proliferation, an absence of myofibroblasts within distal lung and a generalized dysregulation of ECM synthesis. Interestingly, they did not observe any defects in abdominal wall formation or the vasculature of the lung suggesting the mechanisms between these models may be subtly different. This is further highlighted by apparent differences in lung myofibroblasts between these models. Habermehl et al. report a significant decrease in distal myofibroblast numbers at E18.5 and propose that differentiation of myofibroblasts may be attenuated, resulting in the dysregulation ECM required for alveolarization. In contrast, our findings, examining mesenchymal cell populations between E16.5–18.5 suggested that myofibroblast numbers remain normal but that they are functionally compromised. This hypothesis is supported by our in vitro data and existing studies that confirm GCs induce elastin and inhibit collagen synthesis within mesenchymal cell populations [23], [24], [25], [28], [29]. Despite this, both studies suggest an important role for GC mediated ECM synthesis by myofibroblasts in the normal embryonic development of the lung.

### Defective Differentiation of Alveolar Epithelial Cells in GR^Dermo1^ Mice

This study has demonstrated that deletion of mesenchymal GR expression results in cell-non-autonomous effects, inhibiting the differentiation of alveolar epithelial cells. This was characterized by reduced numbers of normal mature AECI and AECII and increased numbers of undifferentiated epithelia in the lungs of GR^Dermo1^ mice. Interestingly, the epithelial phenotype in the GR^Dermo1^ mouse shows similarities to that observed in the epithelial GR null mouse. In particular we saw an identical decrease in expression of the glucocorticoid-regulated epithelial markers SPA, SPB and AQP5 [10], [11]. While GR expression was normal in lung epithelia of GR^Dermo1^ mice we observed a significant decrease in the rapid glucocorticoid response protein GILZ in lung epithelia of GR^Dermo1^ embryos, suggesting downstream glucocorticoid signaling was attenuated. One possible explanation for this observation could be a reduction in local glucocorticoid availability. Therefore, we examined the candidate gene 11β-HSD1 as a measure of glucocorticoid metabolism within lung homogenates of GR^Dermo1^ mice. The enzyme 11β-HSD1 contributes to local glucocorticoid levels within tissues and has been shown to be present within mesenchymal cells, where its expression is positively regulated by glucocorticoids [30], [31]. In the lungs of GR^Dermo1^ mice, 11β-HSD1 mRNA expression was significantly reduced, which may indicate that local glucocorticoid levels are reduced within this tissue. As a consequence, reduced glucocorticoid levels within the lungs of GR^Dermo1^ mice may result in decreased glucocorticoid dependant epithelial differentiation. Alternatively, it may be that mesenchymal cells regulate epithelial differentiation via release of specific glucocorticoid dependant signaling factors, or that elastin itself may have effects on epithelial differentiation.

### Abdominal Wall Defect in GR^Dermo1^ Mice

In this study we have identified a novel role for mesenchymal glucocorticoid signaling in embryonic ventral wall formation: GR^Dermo1^ mice presented with visible intestinal herniation at birth. Furthermore, we observed a marked disruption of elastin and collagen synthesis within the connective tissues adjacent to the herniation. In primary cultures of newborn fibroblasts isolated from tissue homogenates we identified positive transcriptional regulation of type 1 collagen and elastin by glucocorticoids. Although we were not able to examine these regulatory mechanisms in primary cultures from E16.5–E18.5 tissues, these findings certainly demonstrate that glucocorticoids influence mesenchymal ECM synthesis. Other studies have reported decreased transcriptional regulation of collagen type 1 by glucocorticoids in embryonic fibroblasts suggesting that its regulation possess some plasticity [28], [29]. Ultimately, these findings imply an important role for glucocorticoid signaling in regulating ECM components by mesenchymal stromal cells that are required for embryonic ventral wall formation.

Elastins, as well as collagen, have an important role within skin and connective tissues, maintaining their structure and function [32], [33]. Both of these stromal derived extracellular matrix components were significantly reduced in the peri-umbilical region of GR^Dermo1^ mice. Parallel to lung, these changes may reflect direct GR-regulated effects on stromal cell populations. Interestingly, human studies have identified that lung hypoplasia can complicate exomphalos, supporting the concept that the mechanisms responsible for the ventral wall defect and lung atelectasis in the GR^Dermo1^ mice may share a similar etiology [34].

The exact mechanism whereby glucocorticoid-signaling in mesenchymal cells regulates the internalization of the intestines is unclear, although evidence suggests that dysregulated collagen synthesis by mesenchymal fibroblasts contributes to increased risk of herniation as a result of a loss of structural integrity in the connective tissues [35]. Certainly we see a similar dysregulation of key mesenchymal extracellular matrix components such as elastin and collagen in the GR^Dermo1^ mice, which will undoubtedly affect their connective tissue integrity and contribute to the observed abdominal wall defect.

### Conclusions

This study highlights the importance of mesenchymal glucocorticoid signaling in embryonic development. Whilst glucocorticoids have been shown to be important in the maturation and normal function of alveolar epithelia, our findings demonstrate that mesenchymal glucocorticoid signaling possesses a greater association with postnatal lethality [4], [10], [18]. Our data suggest a strong factor contributing to this may be the actions of glucocorticoids on myofibroblast functionality and extracellular matrix synthesis. Consequently, the importance of mesenchymal steroid signaling in embryonic lung and abdominal development provides new insights into the exact roles of glucocorticoids during development.

## Supporting Information

Figure S1 **Supplementary data.** S1A, lung weights in WT and GR^Dermo1^ mice. S1B, IHC staining for fibronectin in pulmonary sections of WT and GR^Dermo1^ mice at E18.5. All results are representative of 5 WT and 5 GR^Dermo1^ mice. SAC = saccule. Scale bar: 20 µM.
(TIFF)

## References

[1] Beato M, Herrlich P, Schutz G (1995) Steroid hormone receptors: many actors in search of a plot. Cell 83: 851–857. 8521509 10.1016/0092-8674(95)90201-5

[2] Parker MG (1993) Steroid and related receptors. Curr Opin Cell Biol 5: 499–504. 8352968 10.1016/0955-0674(93)90016-j

[3] Mangelsdorf DJ, Thummel C, Beato M, Herrlich P, Schutz G, et al (1995) The nuclear receptor superfamily: the second decade. Cell 83: 835–839. 8521507 10.1016/0092-8674(95)90199-x PMC6159888

[4] Cole TJ, Blendy JA, Monaghan AP, Krieglstein K, Schmid W, et al (1995) Targeted disruption of the glucocorticoid receptor gene blocks adrenergic chromaffin cell development and severely retards lung maturation. Genes Dev 9: 1608–1621. 7628695 10.1101/gad.9.13.1608

[5] Speirs HJL, Seckl JR, Brown RW (2004) Ontogeny of glucocorticoid receptor and 11 beta-hydroxysteroid dehydrogenase type-1 gene expression identifies potential critical periods of glucocorticoid susceptibility during development. Journal of Endocrinology 181: 105–116. 15072571 10.1677/joe.0.1810105

[6] Thompson A, Han VK, Yang K (2004) Differential expression of 11beta-hydroxysteroid dehydrogenase types 1 and 2 mRNA and glucocorticoid receptor protein during mouse embryonic development. J Steroid Biochem Mol Biol 88: 367–375. 15145446 10.1016/j.jsbmb.2003.12.014

[7] Bird AD, Tan KH, Olsson PF, Zieba M, Flecknoe SJ, et al (2007) Identification of glucocorticoid-regulated genes that control cell proliferation during murine respiratory development. The Journal of physiology 585: 187–201. 17901120 10.1113/jphysiol.2007.136796 PMC2375468

[8] Bolt RJ, van Weissenbruch MM, Lafeber HN, Delemarre-van de Waal HA (2001) Glucocorticoids and lung development in the fetus and preterm infant. Pediatric pulmonology 32: 76–91. 11416880 10.1002/ppul.1092

[9] Aszterbaum M, Feingold KR, Menon GK, Williams ML (1993) Glucocorticoids Accelerate Fetal Maturation of the Epidermal Permeability Barrier in the Rat. Journal of Clinical Investigation 91: 2703–2708. 8514877 10.1172/JCI116509 PMC443334

[10] Habermehl D, Parkitna JR, Kaden S, Brugger B, Wieland F, et al (2011) Glucocorticoid Activity during Lung Maturation Is Essential in Mesenchymal and Less in Alveolar Epithelial Cells. Mol Endocrinol 25: 1280–1288. 21659474 10.1210/me.2009-0380 PMC5417239

[11] Manwani N, Gagnon S, Post M, Joza S, Muglia L, et al (2010) Reduced viability of mice with lung epithelial-specific knockout of glucocorticoid receptor. Am J Respir Cell Mol Biol 43: 599–606. 20042713 10.1165/rcmb.2009-0263OC PMC5459527

[12] Tronche F, Kellendonk C, Reichardt HM, Schutz G (1998) Genetic dissection of glucocorticoid receptor function in mice. Current opinion in genetics & development 8: 532–538. 9794823 10.1016/s0959-437x(98)80007-5

[13] Li L, Cserjesi P, Olson EN (1995) Dermo-1: a novel twist-related bHLH protein expressed in the developing dermis. Developmental Biology 172: 280–292. 7589808 10.1006/dbio.1995.0023

[14] Yu K, Xu J, Liu Z, Sosic D, Shao J, et al (2003) Conditional inactivation of FGF receptor 2 reveals an essential role for FGF signaling in the regulation of osteoblast function and bone growth. Development 130: 3063–3074. 12756187 10.1242/dev.00491

[15] Baschant U, Frappart L, Rauchhaus U, Bruns L, Reichardt HM, et al (2011) Glucocorticoid therapy of antigen-induced arthritis depends on the dimerized glucocorticoid receptor in T cells. Proceedings of the National Academy of Sciences of the United States of America 108: 19317–19322. 22084093 10.1073/pnas.1105857108 PMC3228447

[16] Schneider JE, McAteer MA, Tyler DJ, Clarke K, Channon KM, et al (2004) High-resolution, multicontrast three-dimensional-MRI characterizes atherosclerotic plaque composition in ApoE−/− mice ex vivo. Journal of magnetic resonance imaging : JMRI 20: 981–989. 15558571 10.1002/jmri.20211

[17] Mariani TJ, Sandefur S, Pierce RA (1997) Elastin in lung development. Experimental lung research 23: 131–145. 9088923 10.3109/01902149709074026

[18] Cole TJ, Solomon NM, Van Driel R, Monk JA, Bird D, et al (2004) Altered epithelial cell proportions in the fetal lung of glucocorticoid receptor null mice. Am J Respir Cell Mol Biol 30: 613–619. 14578211 10.1165/rcmb.2003-0236OC

[19] Williams MC, Cao Y, Hinds A, Rishi AK, Wetterwald A (1996) T1 alpha protein is developmentally regulated and expressed by alveolar type I cells, choroid plexus, and ciliary epithelia of adult rats. American journal of respiratory cell and molecular biology 14: 577–585. 8652186 10.1165/ajrcmb.14.6.8652186

[20] Eddleston J, Herschbach J, Wagelie-Steffen AL, Christiansen SC, Zuraw BL (2007) The anti-inflammatory effect of glucocorticoids is mediated by glucocorticoid-induced leucine zipper in epithelial cells. The Journal of allergy and clinical immunology 119: 115–122. 17208592 10.1016/j.jaci.2006.08.027

[21] Starcher BC (2000) Lung elastin and matrix. Chest 117: 229S–234S. 10.1378/chest.117.5_suppl_1.229s-a 10843923

[22] Wendel DP, Taylor DG, Albertine KH, Keating MT, Li DY (2000) Impaired distal airway development in mice lacking elastin. American Journal of Respiratory Cell and Molecular Biology 23: 320–326. 10970822 10.1165/ajrcmb.23.3.3906

[23] Del Monaco M, Covello SP, Kennedy SH, Gilinger G, Litwack G, et al (1997) Identification of novel glucocorticoid-response elements in human elastin promoter and demonstration of nucleotide sequence specificity of the receptor binding. The Journal of investigative dermatology 108: 938–942. 9182826 10.1111/1523-1747.ep12295241

[24] Mecham RP, Morris SL, Levy BD, Wrenn DS (1984) Glucocorticoids stimulate elastin production in differentiated bovine ligament fibroblasts but do not induce elastin synthesis in undifferentiated cells. The Journal of biological chemistry 259: 12414–12418. 6490622

[25] Swee MH, Parks WC, Pierce RA (1995) Developmental regulation of elastin production. Expression of tropoelastin pre-mRNA persists after down-regulation of steady-state mRNA levels. The Journal of biological chemistry 270: 14899–14906. 7797468 10.1074/jbc.270.25.14899

[26] Pierce RA, Mariani TJ, Senior RM (1995) Elastin in lung development and disease. Ciba Foundation symposium 192: 199–212; discussion 212–194. 10.1002/9780470514771.ch11 8575258

[27] Faury G, Pezet M, Knutsen RH, Boyle WA, Heximer SP, et al (2003) Developmental adaptation of the mouse cardiovascular system to elastin haploinsufficiency. The Journal of clinical investigation 112: 1419–1428. 14597767 10.1172/JCI19028 PMC228452

[28] Perez JR, Shull S, Gendimenico GJ, Capetola RJ, Mezick JA, et al (1992) Glucocorticoid and retinoid regulation of alpha-2 type I procollagen promoter activity. Journal of Cellular Biochemistry 50: 26–34. 1429872 10.1002/jcb.240500107

[29] Oikarinen AI, Vuorio EI, Zaragoza EJ, Palotie A, Chu ML, et al (1988) Modulation of collagen metabolism by glucocorticoids. Receptor-mediated effects of dexamethasone on collagen biosynthesis in chick embryo fibroblasts and chondrocytes. Biochemical pharmacology 37: 1451–1462. 3358778 10.1016/0006-2952(88)90006-8

[30] Hardy RS, Filer A, Cooper MS, Parsonage G, Raza K, et al (2006) Differential expression, function and response to inflammatory stimuli of 11beta-hydroxysteroid dehydrogenase type 1 in human fibroblasts: a mechanism for tissue-specific regulation of inflammation. Arthritis research & therapy 8: R108. 16846535 10.1186/ar1993 PMC1779419

[31] Ahasan MM, Hardy R, Jones C, Kaur K, Nanus D, et al.. (2012) Inflammatory regulation of glucocorticoid metabolism in mesenchymal stromal cells. Arthritis and rheumatism. 10.1002/art.34414 PMC3532601 22294469

[32] Cavalcante FS, Ito S, Brewer K, Sakai H, Alencar AM, et al (2005) Mechanical interactions between collagen and proteoglycans: implications for the stability of lung tissue. Journal of applied physiology 98: 672–679. 15448123 10.1152/japplphysiol.00619.2004

[33] Tiganescu A, Walker EA, Hardy RS, Mayes AE, Stewart PM (2011) Localization, age- and site-dependent expression, and regulation of 11beta-hydroxysteroid dehydrogenase type 1 in skin. The Journal of investigative dermatology 131: 30–36. 20739946 10.1038/jid.2010.257

[34] Vachharajani AJ, Rao R, Keswani S, Mathur AM (2009) Outcomes of exomphalos: an institutional experience. Pediatric surgery international 25: 139–144. 19066916 10.1007/s00383-008-2301-y

[35] Friedman DW, Boyd CD, Norton P, Greco RS, Boyarsky AH, et al (1993) Increases in type III collagen gene expression and protein synthesis in patients with inguinal hernias. Annals of surgery 218: 754–760. 7710461 10.1097/00000658-199312000-00009 PMC1243071

