# Supplementary figures and images for: Deletion of Mesenchymal Glucocorticoid Receptor Attenuates Embryonic Lung Development and Abdominal Wall Closure
Source: PLoS One. 2013 May 16;8(5):e63578. doi: 10.1371/journal.pone.0063578 (PMC3656055; doi:10.1371/journal.pone.0063578)

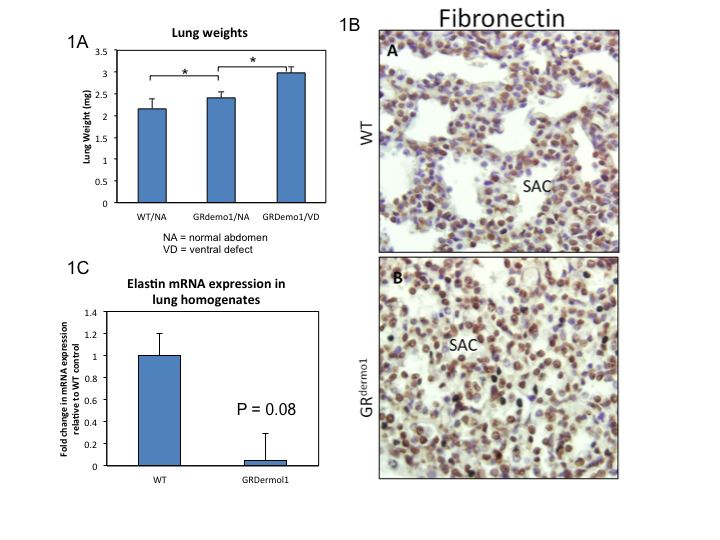

Supplement: Figure S1 — Supplementary data. S1A, lung weights in WT and GRDermo1 mice. S1B, IHC staining for fibronectin in pulmonary sections of WT and GRDermo1 mice at E18.5. All results are representative of 5 WT and 5 GRDermo1 mice. SAC = saccule. Scale bar: 20 µM. (TIFF) [file pone.0063578.s001.tiff]
